# Computed tomography scan usage when US results are non-diagnostic for suspected acute appendicitis in children

**DOI:** 10.1097/MD.0000000000021961

**Published:** 2020-09-04

**Authors:** Injoon Kim, Hyuksool Kwon, Yoo Jin Choi, Young Ho Kwak, Jin Hee Lee, Dongbum Suh, Jae Yun Jung, Joong Wan Park

**Affiliations:** a Department of Emergency Medicine, Seoul National University Hospital, Seoul; b Department of Emergency Medicine, Seoul National University Bundang Hospital, Seongnam; c Department of Emergency Medicine, Ajou University School of Medicine, Suwon, Republic of Korea.

**Keywords:** Alvarado's appendicitis score, appendicitis, computed tomography, false negative, pediatric, ultrasound

## Abstract

This retrospective study was aimed to determine the factors suggesting the need for computed tomography (CT) scanning when ultrasound (US) imaging results are negative or non-diagnostic in children suspicious for acute appendicitis in the emergency department.

Patients less than 18 years old who underwent abdominal ultrasound and CT to rule out acute appendicitis were enrolled. Patients were classified into 2 groups: the false-negative group, in which patients had negative or non-diagnostic results on the initial US and a final diagnosis of acute appendicitis on the following abdominal CT, and the true-negative group, in which patients had negative or non-diagnostic US results and were negative on abdominal CT. Logistic regression and propensity score matching with the predicting factors were performed.

The presence of vomiting (odds ratio (OR), 7.78; 95% confidence interval (CI), 1.92–41.04) and poor oral intake (OR, 4.67; 95% CI, 1.21–21.15) with a high white blood cell (WBC) count (OR 1.26; 95% CI, 1.09–2.37), segmented neutrophil ratio (OR, 1.09; 95% CI, 1.03–1.16), and C-reactive protein (CRP) (OR, 1.49; 95% CI, 1.09–2.37) were suggestive of the false-negative group. The propensity-matched population also showed significant associations with vomiting (OR, 7.86; 95% CI, 1.65–37.40) and poor oral intake (OR, 5.50; 95% CI, 1.28–23.69) with an elevated WBC count (OR, 1.27; 95% CI, 1.08–1.50), segmented neutrophil ratio (OR, 1.09; 95% CI, 1.03–1.16), and CRP (OR, 1.51; 95% CI, 1.03–2.22).

A CT scan should be considered in children with suspected acute appendicitis if they have vomiting, high CRP, and high WBC count, despite negative or non-diagnostic US results.

## 1 Introduction

Acute appendicitis is the most common surgical emergency in the pediatric population worldwide.^[1]^ Appendicitis, however, is often challenging to diagnose due to its varied presentation in terms of symptoms, signs, and predictive laboratory values. Particularly in the pediatric population, diagnosis is more difficult due to the communication barrier and poor coordination during abdominal examinations.^[2]^ Thus, the diagnosis may be delayed or even missed at initial presentation, possibly leading to complications such as perforation, abscess formation, and peritonitis and resulting in increased morbidity and mortality.^[3,4]^ Therefore, rapid and accurate diagnosis of appendicitis is critical in pediatric patients.

While low-dose abdominal computed tomography (CT) scan is the modality of choice for diagnosis of acute appendicitis in adult patients in recent years,^[5]^ ultrasonography (US) is usually the initial diagnostic modality in young children due to the risk of radiation exposure during CT scanning.^[6]^ US, however, cannot safely exclude appendicitis, especially in high-risk patients, even if the US result is negative.^[7]^ Several studies attempted to find the risk factors for diagnostic error in US.^[8,9]^ Although a previous study proposed a “staged US and CT” protocol,^[10]^ there is no specific guideline for the staged CT protocol for pediatric patients with negative or non-diagnostic results on US.

We conducted this study to find factors associated with false negative or non-diagnostic appendicitis cases in the US, necessitating further evaluation with CT.

## 2 Method

### 2.1 Study design and setting

This retrospective case-control study was performed at an urban tertiary care hospital with 1,100 beds and approximately 92,000 emergency department (ED) visits annually. The pediatric radiologists perform more than 400 cases of pediatric abdominal ultrasonography with more than 40 children with acute appendicitis presenting annually, and about 82% of the patients were diagnosed with US, and the others were with CT. Concerning the radiation hazards, patients with suspected acute appendicitis under 15 years of age were first evaluated by US in this institution. The CT scan protocol for the young patients were limited to low-dose, single phase CT to reduce radiation exposure.

The medical record review was performed by 2 independent researchers. The Seoul National University Bundang Hospital Institutional Review Board approved the protocol and analysis plan (IRB No: B-1909-567-101). As the study was designed as retrospective observational study, the need for informed consent was waived by the review board.

### 2.2 Participants

The study included patients under 18 years of age who visited the ED with suspected appendicitis and underwent US followed by abdominal CT to rule out acute appendicitis from April 1, 2014 to March 31, 2018. Patients with apparent acute appendicitis on initial US and those who did not undergo an abdominal CT scan were excluded.

### 2.3 Definition and classification

Enrolled patients were classified into 2 groups:

(1) the false-negative group, in which patients had negative or non-diagnostic results on initial US studies and a final diagnosis of acute appendicitis on abdominal CT;
(2) the true-negative group, in which patients had negative or non-diagnostic US and abdominal CT results.

Negative results were defined as a fully visualized normal appendix, with or without other apparent pathologic findings. Other results, including poor visualization, any non-visualization reports, including cecal connection, tip, or whole appendix, were considered non-diagnostic.

### 2.4 Data collection

For each medical record reviewed, the following data were collected: age; sex; body weight; date and time of ED visit; date and time of US examination; US reports; degree of radiologists (faculty or residents); symptoms, including abdominal pain, febrile sense, lethargy, vomiting, diarrhea, constipation, poor oral intake, and migration of pain; signs, including documented fever more than 38 degrees centigrade, right lower quadrant tenderness, and any peritoneal signs; laboratory findings, including elevated white blood cell (WBC) count, segmented neutrophil ratio, and c-reactive protein (CRP); and CT reports, including the CT appendicitis score. We also calculated Alvarado's appendicitis score^[11]^ for each case. The well-known scoring system has been validated in both adult and pediatric populations.^[12–14]^

### 2.5 Study outcome

The primary outcome of this study was to determine the factors suggestive of diagnostic errors, including false negatives and non-diagnostic results in children with acute appendicitis. The secondary outcome was to compare the final model of this study with previously established prediction models, such as the Alvarado's appendicitis score used in this study.

### 2.6 Data analysis

We calculated descriptive statistics for all variables of interest. Demographics of the 2 groups were compared using a Chi-square test or *t*-test where applicable. Statistical significance was set at a *P*-value of .05. We performed univariable analysis using demographic, clinical, and laboratory findings to determine the factors suggestive of a false-negative group. We then performed multivariable logistic regression and propensity score matching with the predicting factors derived from the univariable analysis. Propensity matching was done using the nearest matching method. The receiver operating characteristic (ROC) curve of the final model of logistic regression and the Alvarado's appendicitis score model were compared using DeLong's test. We further evaluated diagnostic accuracy of the final model and Alvarado's appendicitis score by calculating specificity, sensitivity, positive predictive value and negative predictive value. Statistical analysis was performed using R x64 Version 3.6.0.

## 3 Results

**Figure 1:**
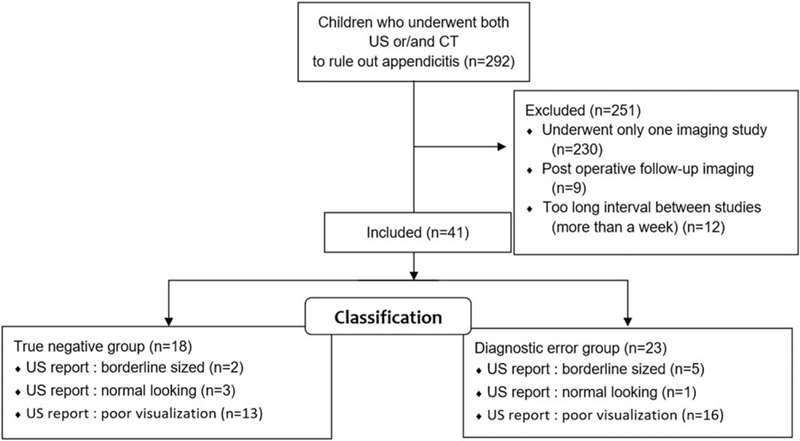
Enrollment and classification. US = ultrasound; CT = computed tomography.

From April 1, 2014 to March 31, 2018, 292 children who underwent US, CT, or both to rule out acute appendicitis were identified. Of the 292 patients, 41 patients (14%) experienced both studies. Among the 41 patients, 18 patients comprised the true-negative group, while 23 were in the false-negative group (Fig. 1).

There was no significant difference between the 2 groups regarding demographic characteristics such as sex, age, and body weight (Table 1). The time of US examination, categories of non-diagnostic reports, and training status of radiologists were not significantly different between groups. Vomiting was associated with the false-negative group (17% vs 61%, *P* = .01). Fever, constipation, diarrhea, and lethargy were not more greatly associated with a certain group. Any physical sign of acute appendicitis was not significantly different between the 2 groups. The false-negative group had higher WBC count (mean value 9,700/μL vs 15,700/μL, *P* = .001), segmented neutrophil ratio (mean value 59.8% vs 77.8%, *P* < .001), and CRP (mean value 0.3 mg/dL vs 2.4 mg/dL, *P* = .02). The CT appendicitis score was significantly higher in the false-negative group. Most had a score of 5 (0% vs 83%), indicating perforated appendicitis.

**Table 1:**
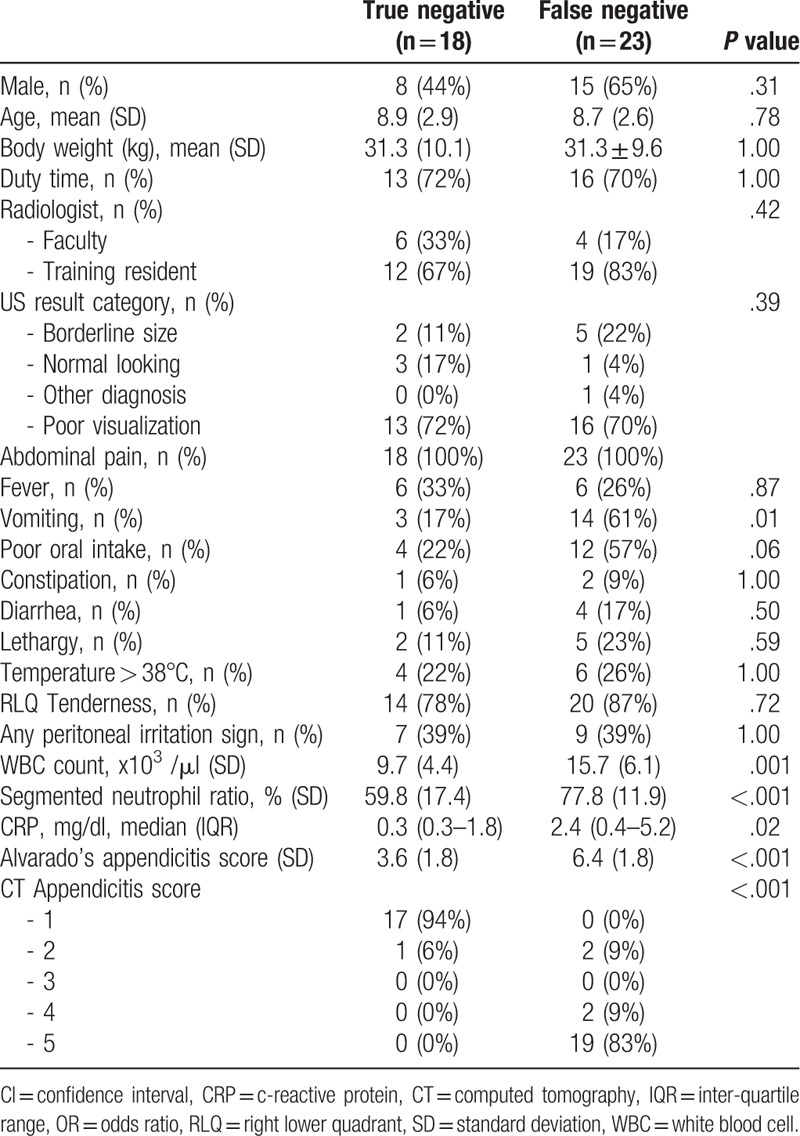
Baseline characteristics.

On univariable analysis, the false-negative group was more likely to have vomiting (OR, 7.78; 95% CI, 1.92–41.04), poor oral intake (OR, 4.67; 95% CI, 1.21–21.15). WBC (OR, 1.26; 95% CI, 1.09–2.37), segmented neutrophil ratio (OR, 1.09; 95% CI, 1.03–1.16), and CRP (OR, 1.49, CI, 1.09–1.37) were significantly higher in the false-negative group (Table 2). Multivariable logistic regression showed that the presence of vomiting (OR, 5.40; 95% CI, 0.91–41.09), high WBC count (OR, 1.14; 95% CI, 0.96–1.39), and high CRP (OR, 1.47; 95% CI, 0.98–2.84) were factors suggestive of the false-negative group (Table 2).

**Table 2:**
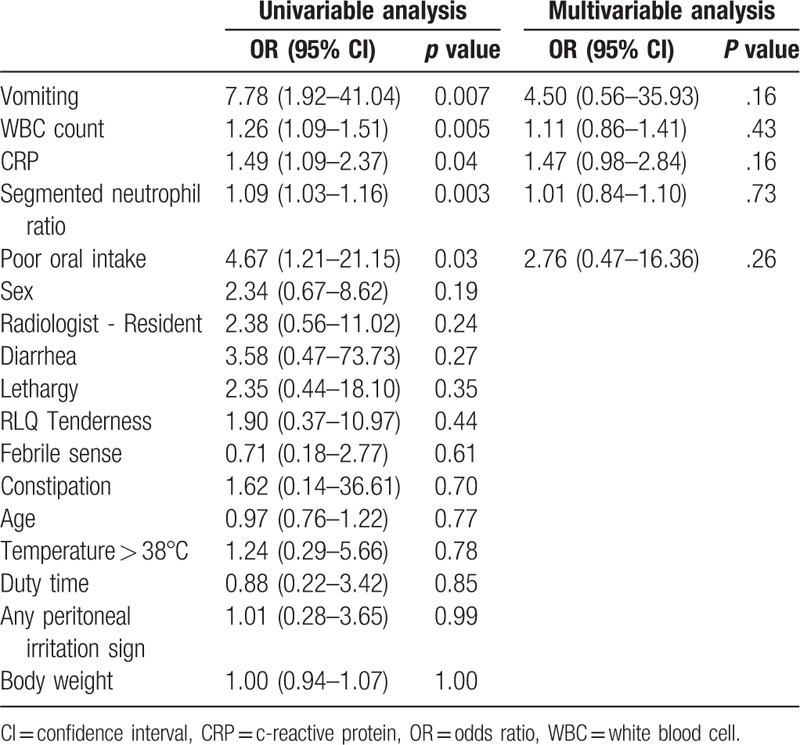
Logistic regression.

Factors identified in the univariable analysis were matched using the nearest matching method. Three cases were excluded in the matching analysis due to missing values. Two cases were unmatched. Eighteen cases in each group were matched. Vomiting (3 [17%] vs 11 [61%], *P* = .02), poor oral intake (4 [22%] vs 11 [61%], *P* = .04), WBC count (9,700/μl?,400 vs 16,400/μl?,300, *P* = .001), segmented neutrophil ratio (59.8?7.4% vs 77.3?2.6%, *P* = .001), and CRP (1.2?.2 mg/dL vs 4.2?.8 mg/dL, *P* = .02) were significantly different between the 2 propensity-matched groups (Table 3). These differences correlate with the result of the univariable analysis of the total population (Table 4).

**Table 3:**
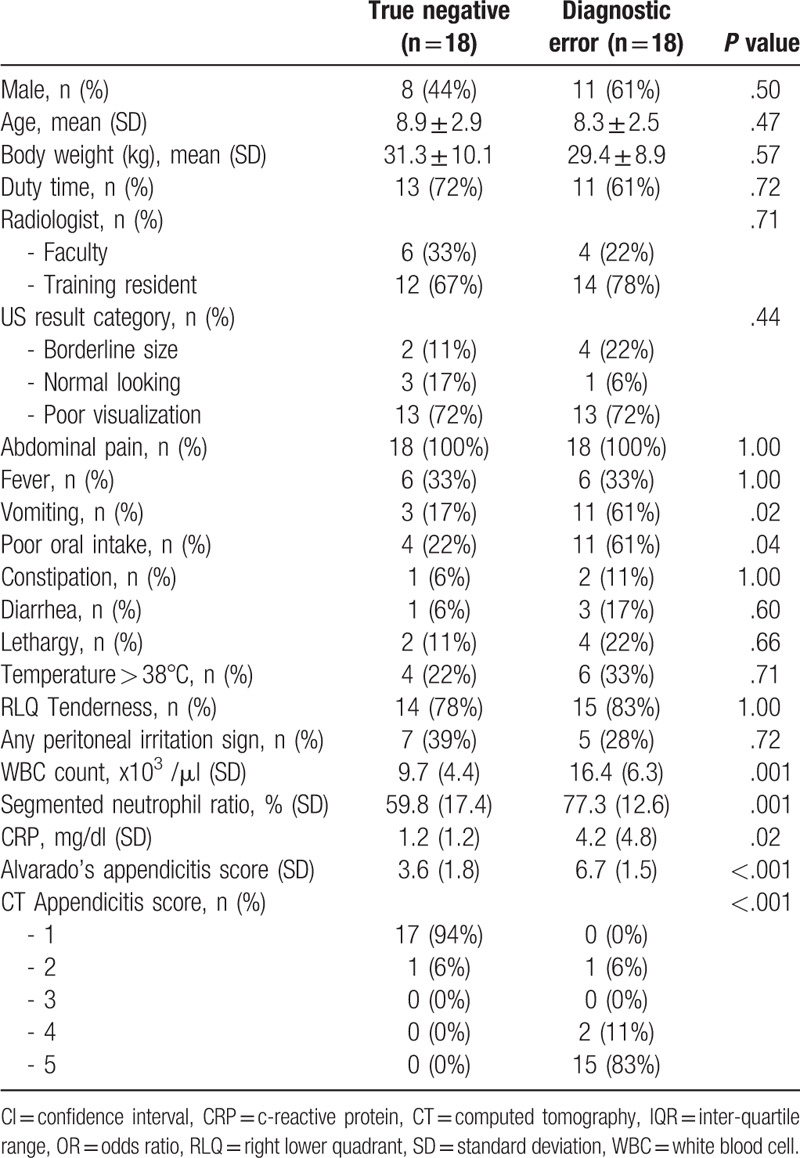
Characteristics of the propensity-matched population.

**Table 4:**
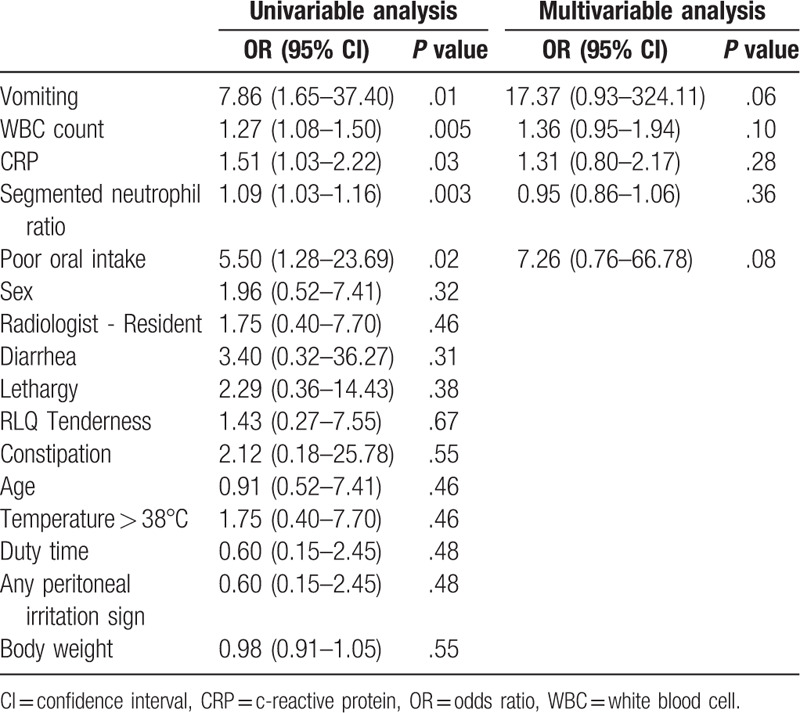
Factors associated with false negatives in ultrasound examination in the propensity-matched population.

**Figure 2:**
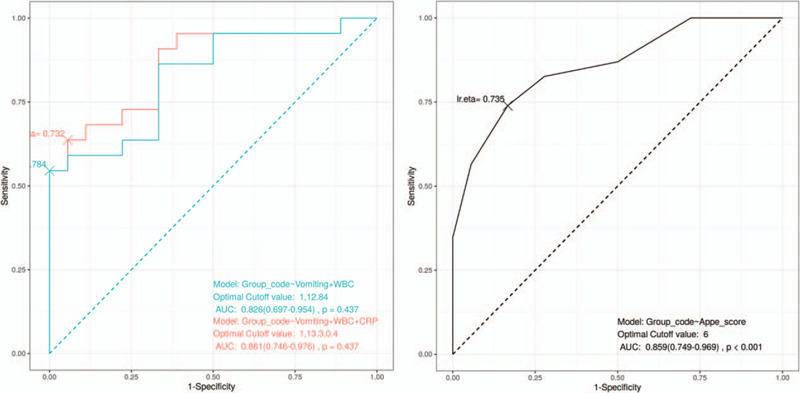
ROC curves of the final model of multivariable logistic regression and Alvarado's appendicitis score.

We compared ROC curves of the final model of the multivariable logistic regression and of Alvarado's appendicitis score (Fig. 2). The ROC curve of the final model had an area under the curve of 0.861. The optimal cut-off value for WBC count and CRP was 13,300/μL and 0.40 mg/dL, respectively. The optimal cut-off value for Alvarado's appendicitis score model was 6 for distinguishing the false-negative group from the true-negative group, with an area under the curve of 0.859. The 2 ROC curves were not significantly different on DeLong test (*P* = .43).

Using the cut-offs of CRP (0.40 mg/dL), WBC (13,300/μL) and the presence of vomiting, the combination of the factors showed sensitivity of 0.39, and specificity of 1.00 (Table 5). Positive predictive value was 1.00 and negative predictive value was 0.56. If the combination were applied to the participants, CT scans could have been reduced in false negative group by 100%. On the other hand, Alvarado's appendicitis score showed sensitivity of 0.74, and specificity of 0.83, showing relatively higher sensitivity than the final model of this study. Positive predictive value was 0.85, and negative predictive value was 0.71.

**Table 5:**
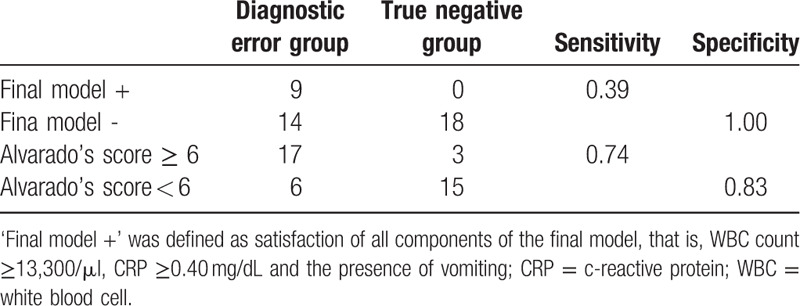
Diagnostic accuracy of the final of multivariable logistic regression and Alvarado's appendicitis score.

## 4 Discussion

In this study, we observed more frequent vomiting and poor oral intake, higher WBC count, segmented neutrophil count, and CRP in the false-negative group. The optimal cut-off value for the WBC count and CRP was 13,300/μL and 0.40 mg/dL, respectively.

There was no significant difference in demographic characteristics, such as age, sex, and body weight, between the 2 groups. All patients in both groups had abdominal pain. Fever, right lower quadrant tenderness, and peritoneal signs were not associated with the results. Since all enrolled patients were suspected of having appendicitis, this finding is plausible. This finding suggests that these factors do not help guide the diagnosis of a clinically high-risk patient with negative or non-diagnostic US results.

Conversely, poor oral intake and vomiting were more frequent in the false-negative group. These factors may be associated with the complications of appendicitis, such as paralytic ileus due to intra-abdominal inflammation. For patients with high suspicion of acute appendicitis, CT scans should be taken, especially if the patients have vomiting or poor oral intake with negative or non-diagnostic initial US results. The CT appendicitis score was significantly higher. Most had a score of 5, indicating perforated appendicitis.

The training status of the radiologist and the time of sonographic examination were not associated with the results. Though the performer's competence is important in an ultrasonographic study,^[15]^ diagnostic error was similar regardless of the training status of the performer in clinically high-risk patients.

In patients with non-diagnostic results, most results reported poor visualization. Furthermore, the CT appendicitis score was usually 5 in the diagnostic group and 1 in the true-negative group. A CT appendicitis score of 5 indicates perforated and complicated appendicitis. This finding suggests that the perforated appendicitis is prone to be missed in abdominal US.

We compared our final model with Alvarado's appendicitis score.^[11]^ The scoring system was first described in 1986 and had been validated in both adult and pediatric populations in many previous studies.^[12–14]^ 6 or higher in Alvarado's appendicitis score showed notable sensitivity and specificity in this high-risk population, while the final model of this study showed higher specificity. These 2 predictive factors could be used to determine whether CT scan is needed or not, in individual contexts.

The diagnosis of acute appendicitis in children is challenging. Imaging studies give essential clues for this diagnosis. US is considered the primary diagnostic modality in children despite having lower sensitivity and specificity compared with CT due to its lower risk of radiation exposure.^[15]^ Conclusive US reports indicating acute appendicitis help clinicians in diagnosis, but non-diagnostic results are frequent due to poor visualization or borderline sizing.^[8]^ Even negative reports for acute appendicitis may be false negatives.

Thus, negative and non-diagnostic US results are troublesome for clinicians. For such cases, the clinician may choose to further evaluate using abdominal CT scans or repeat US or to discharge the patient home if the clinical possibility of acute appendicitis is low.^[10]^ Though we cannot completely assure that the patients discharged without further imaging studies truly do not have acute appendicitis, we assume that these patients have a lower possibility than those requiring additional abdominal CT scans. Thus, we excluded the out-of-interest group in this study.

There are several limitations to this study. First, the number of cases enrolled in this study is small. It was a single-center study with complicated conditions for enrollment since only patients with both abdominal US and CT results were included. We compensated for this limitation with the propensity-score matching method. Second, radiation exposure may be increased with more frequent CT scans, as suggested in this study. The results of this study, however, should be considered only after the initial US examination and in clinically high-risk patients with negative or non-diagnostic US reports. Finally, since this study is retrospective, a prospective study is warranted in the future to confirm the factors identified in this study.

## 5 Conclusion

CT scans should be considered in children with high clinical suspicion of acute appendicitis if they have vomiting, high WBC count and elevated serum CRP concentration, despite negative or non-diagnostic US results. Alvarado's appendicitis score is also a useful tool for decision-making in this population.

## Author contributions

**Conceptualization:** Injoon Kim, Hyuksool Kwon.

**Data curation:** Injoon Kim, Hyuksool Kwon, Yoo Jin Choi.

**Formal analysis:** Injoon Kim, Hyuksool Kwon, Yoo Jin Choi.

**Investigation:** Injoon Kim.

**Methodology:** Injoon Kim, Hyuksool Kwon, Yoo Jin Choi, Young Ho Kwak, Jin Hee Lee, Dongbum Suh, Jae Yun Jung, Joong Wan Park.

**Resources:** Hyuksool Kwon, Yoo Jin Choi.

**Supervision:** Hyuksool Kwon, Yoo Jin Choi.

**Writing – original draft:** Injoon Kim.

**Writing – review & editing:** Hyuksool Kwon, Yoo Jin Choi, Young Ho Kwak, Jin Hee Lee.
